# Neural tube defects: Sex ratio changes after fortification with folic acid

**DOI:** 10.1371/journal.pone.0193127

**Published:** 2018-03-14

**Authors:** Fernando A. Poletta, Monica Rittler, Cesar Saleme, Hebe Campaña, Juan A. Gili, Mariela S. Pawluk, Lucas G. Gimenez, Viviana R. Cosentino, Eduardo E. Castilla, Jorge S. López-Camelo

**Affiliations:** 1 Latin American Collaborative Study of Congenital Malformations (ECLAMC) at Center for Medical Education and Clinical Research (CEMIC-CONICET), Buenos Aires, Argentina; 2 ECLAMC at INAGEMP (National Institute of Population Medical Genetics), Rio de Janeiro, Brazil; 3 ECLAMC at Hospital Materno Infantil Ramón Sardá, University of Buenos Aires, Buenos Aires, Argentina; 4 ECLAMC at Maternity Hospital Nuestra Señora de las Mercedes, Tucumán, Argentina; University of Missouri Columbia, UNITED STATES

## Abstract

**Background:**

Historically, neural tube defects (NTDs) have predominated in female infants but the reasons remain unclear. In South America, the pre- folic acid fortification (FAF) rates of NTDs were around 18/10,000 births for females and 12/10,000 births for males, with an estimated sex ratio (male/female) of 0.67. During the post- FAF period, unpublished routine reports have indicated changes in the sex ratio for these defects while some descriptive reports are controversial. To date and to our knowledge, however, no studies specifically focusing on these changes to test this hypothesis directly have been undertaken. The aim of this study was to analyze changes in the sex ratio of infants with NTDs after FAF in South American countries.

**Materials and methods:**

With a descriptive cross-sectional study design, 2,597 infants with isolated NTDs born between 1990 and 2013 in 3 countries participating in the Latin American Collaborative Study of Congenital Malformations (ECLAMC) network were included: (Chile N = 521 and Argentina N = 1,619 [with FAF policies]; Venezuela N = 457 [without FAF policies; used as control]; total births = 2,229,561). The differences-in-differences method and Poisson regressions were used to evaluate the sex ratio shift from female to male before vs. after FAF, and to assess whether these differences were related to the fortification.

**Results and conclusions:**

In Chile and Argentina the prevalence of NTDs, particularly anencephaly and cervico-thoracic spina bifida, showed a greater reduction rate in females than in males after FAF, resulting in a change of the sex ratio of infants with NTDs. Some mechanisms possibly involved in this differential reduction are proposed which might be useful to identify the pathogenesis of NTDs as a whole and specifically of those susceptible to the protective effect of folic acid.

## Introduction

Neural tube defects (NTDs), including spina bifida, anencephaly and cephalocele, are severe birth defects of the central nervous system that occur during embryonic development when the neural tube fails to close completely. In South America before folic acid fortification, as well as in other countries without fortification policies, the overall prevalence of NTDs varied between 10 and 20 per 10,000 births [1], and showed a historical female sex predisposition for the three malformations. The observed frequency in South America was 18/10,000 for females and 12/10,000 for males [2], with an estimated sex ratio (SR = male/female) of 0.67, which however differed among other studies. For the US NBDPS registry [3], it was closer to an equal male/female relationship, while Lary and Paulozzi [4], Shaw et al. [5], and Rittler et al. [2] found a greater female excess. Epigenetic phenomena [6], a relatively higher proportion of males with associated NTDs and lost during early gestation [7], and/or differences in susceptibility to environmental factors, based on the multifactorial theory [8], could explain the female excess.

Based on the evident reduction of NTDs after periconceptional folic acid fortification (FAF) [9–12], three countries in South America have implemented national fortification policies: Chile in January 2000 [13], Argentina in December 2003 [14], and Brazil in July 2004 [15]. While Chile and Argentina have fortified wheat flour with 2.2 mg/kg to provide an estimated average of 400 μg/day of folic acid as recommended by the Centre for Disease Control [16]; Brazil has fortified wheat and corn flour at a lower dose (1.5mg/kg) [15, 17]. Thus, the estimated average of daily ingested folic acid was 499 μg in Chile [13], 486 μg in Argentina [14, 18], and 264 μg in Brazil [15]. In previous studies we have shown the impact of these policies: in Chile and Argentina the observed NTD prevalence declined to almost a half after folic acid fortification [1, 12], while in Brazil such a strong reduction could not be as clearly observed, showing more heterogeneous rates [1, 17].

During the post- FAF period, unpublished routine reports have indicated changes in the sex ratio for these defects while some descriptive reports are controversial [19–20]. To date and to our knowledge, however, no studies specifically focusing on these changes to test this hypothesis directly have been undertaken.

The purpose of the present work was to analyze eventual changes in the SR of NTDs after folic acid fortification in the two South American countries, Chile and Argentina, where a clear effectiveness of fortification on the reduction of NTDs has been verified.

## Materials and methods

By using a descriptive cross-sectional study, 2,597 live and stillborn infants with isolated NTDs among 2,229,561 total births (TB) from 95 maternity hospitals of 3 South American countries participating in the Latin American Collaborative Study of Congenital Malformations (ECLAMC)[21–22] between 1990 and 2013, were analyzed: (Chile N = 521, TB = 565,238; Argentina N = 1619, TB = 1,310,367; Venezuela N = 457, TB = 353,956). Venezuela, where no fortification policy has been implemented, was used as control group.

ECLAMC is a program dedicated to the research of birth defects, through a network of maternity hospitals where health professionals, mainly pediatricians, identify birth defects in live and stillborn infants. Data on socioeconomic and demographic characteristics, previous birth outcomes, and prenatal factors are obtained from medical records and by interviewing the mothers of malformed infants and of healthy controls, before their discharge. Detailed descriptions of the registry and methodology have been published previously [21–22]. Written and signed informed consents were obtained for all subjects participating in the ECLAMC program before data collection. Furthermore, ECLAMC pediatricians adequately explain the written informed consent content to the mother or legal guardian of the newborn. The study protocol was approved by the Ethics Committee “Center for Medical Education and Clinical Research (CEMIC)” (DHHS-IRB #1745, IORG #1315), and all written consent are available in the ECLAMC coordination headquarters.

The NTD sample included the following anomalies: anencephaly, spina bifida total and subtypes (cervico-thoracic or high, and lumbo-sacral or low), and cephalocele. Since terminations of pregnancies are illegal in these countries, they were excluded.

Two approaches were used:

1. Comparison of the SR by NTD type, before vs. after FAF and between countries with vs. without FAF; 2) comparison of the NTD reduction rates between females and males.

For the first approach, the secular trend of the SR was established to determine if changes had occurred during the pre-FAF period; for this step, a linear regression model was used. The independent variables included dummy variables to represent the time periods before FAF and dummy hospitals as fixed effects to control heterogeneity among them.

To confirm whether the SR had changed after FAF, we used the differences-in-differences (DID) method [23], a quasi-experimental technique used to measure the effect of a treatment over a specific time period with treatment and control groups. Chile and Argentina were considered as treatment groups, Venezuela as control group.

The DID estimator was used representing the difference between the pre- and post-FAF measures in the treatment (Chile and Argentina) and control (Venezuela) groups and the pre- and post-FAF periods representing the difference between periods. Chile began fortifying in 2000; therefore, the NTD cases were grouped by dates of birth into two periods (before 2000 and after 2001). Similarly, Argentina began fortifying at the end of 2003, so the periods were defined as before 2004 and after 2005. For Venezuela, without fortification, the periods were defined with the same dates as those of the country to which it was being compared.

For two periods (before and after FAF) and two groups (Venezuela: control group and Chile/Argentina: treatment group), Y_11_ and Y_12_ represented the SR change in the control group before and after FAF, respectively, and Y_21_ and Y_22_ represented the SR change before and after FAF in the treatment group.

The DID estimator was expressed as: (Y_22_–Y_21_)-(Y_12_-Y_11_).This estimator measured the SR changes in the post-FAF period of the treatment group compared with the SR changes in the control group. Under the alternative hypothesis, the sex ratio changes in the post-FAF period/treatment group should be Y_12_-Y_11_ = 0, Y_22_>Y_21_ and DID>0. The Monte Carlo simulation was used to calculate the standard error of the DID estimator.

For the second approach, we used a Poisson regression to assess the NTD prevalence reduction after FAF, with the following model:

Log(n)=α+aSex+bFA+cSexFA+∑Hj;

where n is the number of NTD cases (male or female), Sex is a dummy variable representing the female (sex = 1) and male (sex = 0) cases, FA is a dummy variable for pre-FAF (fa = 0) and post-FAF (fa = 1) periods, SexFa is an interaction term, where c is a coefficient that assesses NTD reduction rate differences between females and males, and H is the dummy variable for j hospitals. Hospitals were included in the model as fixed effects to minimize biases by unobserved variables that may differ among hospitals. The Huber method [24] was used to estimate the standard errors controlling the intergroup correlations because the same hospitals were included in the model for different years.

This sample size allowed us to detect a minimum change of 25% in the SR for an 80% of power (β = 0.20) and a 5% of type I error (α <0.05).

An entire database including raw data of number of cases and total births tabulated by country, year, hospital and sex; as well as a complete description of database structure and variables, are available as Supporting Information Files (S1 and S2, respectively).

## Results

**Fig 1:**
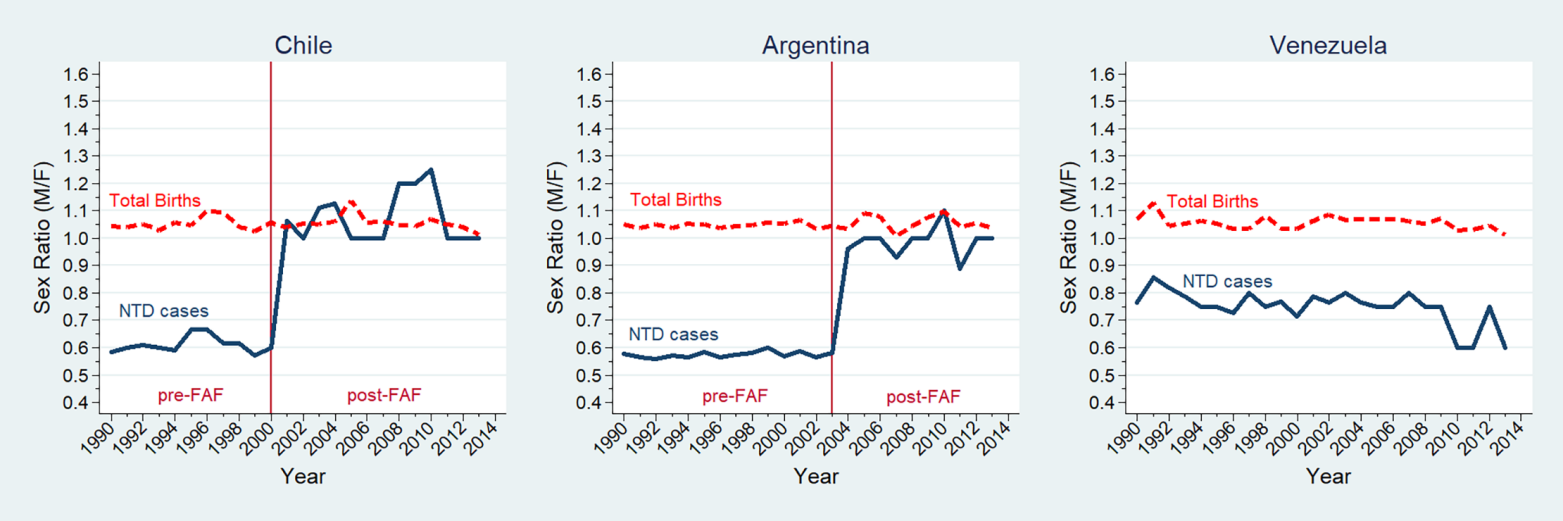
Sex ratio changes for NTD cases and total births in Chile, Argentina, and Venezuela (1990–2013). NTD: neural tube defect; FAF: folic acid fortification; M/F: male/female; Sex ratio (male/female) for neural tube defect cases (full blue line), sex ratio for total births (dashed red line). Sex ratios estimated by multivariate regression models adjusted by hospital.

The number of cases by anomaly type, country, gender, and FAF period are shown in Table 1. The total number of births with specified sex was 2,229,561 (98.6%), and the population SR (M/F) of the 3 countries did not change during the whole study period (Chile: before FAF = 1.051, after FAF = 1.055, p = 0.1758; Argentina: before FAF = 1.041, after FAF = 1.049, p = 0.192; and Venezuela = 1.061) (Fig 1).

**Table 1:** Number of NTD cases by period (pre- and post- FAF) and sex of newborns.

		Pre FAF		Post FAF		
Country	Defect	Male	Female	Male	Female	Total
CHL	NTD total	125	207	97	92	521
	Anencephaly	52	98	38	44	232
	Spina bifida	58	87	43	38	226
	Cervico-thoracic	14	37	2	2	55
	Lumbo-sacral	44	50	41	36	171
	Cephalocele	15	22	16	10	63
	Total Births	115,127	109,478	174,955	165,678	565,238
ARG	NTD total	489	855	138	137	1619
	Anencephaly	183	437	58	51	729
	Spina bifida	252	322	64	67	705
	Cervico-thoracic	63	107	12	8	190
	Lumbo-sacral	189	215	52	59	515
	Cephalocele	54	96	16	19	185
	Total Births	478,971	459,867	190,197	181,332	1,310,367
VEN	NTD total	197	260	-	-	457
	Anencephaly	65	90	-	-	155
	Spina bifida	120	149	-	-	269
	Cervico-thoracic	44	55	-	-	99
	Lumbo-sacral	76	94	-	-	170
	Cephalocele	12	21	-	-	33
	Total Births	182,256	171,700			353,956

FAF: folic acid fortification; CHL: Chile; ARG: Argentina; VEN: Venezuela.

In Chile and Argentina, the secular trend of the SR in NTD cases was not significant for the pre- FAF period (b = 0.015, p = 0.728; and b = -0.021, p = 0.158; respectively), while in Venezuela it remained stable across the entire period (b = 0.009, p = 0.594) (Fig 1). When compared with Venezuela, a significant increase of the SR in NTD cases pre- vs. post- FAF was observed in Chile (from 0.60 to 1.05) and Argentina (from 0.57 to 1.01) (Table 2).

**Table 2:** Sex ratio (M/F) changes for types of NTDs between FAF periods.

	Sex Ratio (M/F) Chile vs. Venezuela										
	Pre-FAF			Post-FAF							
Defect	CHL	VEN	Diff	CHL	VEN	Diff	DID	95% CI		Z	P
NTD total	0.60	0.65	-0.05	1.05	0.89	0.16	0.21	0.06	0.36	2.61	0.004
Anencephaly	0.53	0.74	-0.21	0.86	0.69	0.18	0.39	0.16	0.61	3.39	0.001
Spina bifida	0.67	1.04	-0.38	1.13	0.59	0.54	0.91	0.69	1.14	7.92	0.001
Cervico-thoracic	0.38	1.03	-0.66	1.00	0.54	0.46	1.12	0.02	2.21	1.99	0.023
Lumbo-sacral	0.88	0.70	0.18	1.14	0.62	0.52	0.34	0.09	0.59	2.66	0.004
Cephalocele	0.68	0.58	0.10	1.60	0.56	1.04	0.95	0.28	1.60	2.80	0.002
Total Births	1.04	1.05	-0.01	1.06	1.06	0.00	0.01	-0.19	0.09	0.45	0.326
	Sex Ratio (M/F) Argentina vs. Venezuela										
	Pre-FAF			Post-FAF							
Defect	ARG	VEN	Diff	ARG	VEN	Diff	DID	95% CI		Z	P
NTD total	0.57	0.74	-0.16	1.01	0.81	0.20	0.36	0.22	0.50	4.95	0.001
Anencephaly	0.42	0.69	-0.27	1.14	0.87	0.27	0.55	0.21	0.87	3.25	0.001
Spina bifida	0.78	0.80	-0.02	0.96	0.81	0.15	0.17	0.07	0.33	2.04	0.020
Cervico-thoracic	0.59	0.86	-0.27	1.50	0.76	0.74	1.00	0.19	1.81	2.41	0.008
Lumbo-sacral	0.88	0.77	0.05	0.88	0.83	0.05	-0.06	-0.25	0.13	-0.60	0.274
Cephalocele	0.56	0.75	-0.19	1.19	0.46	0.73	0.91	0.52	1.30	4.64	0.001
Total Births	1.04	1.06	-0.01	1.06	1.06	0.00	0.01	-0.07	0.12	0.54	0.295

FAF: folic acid fortification; NTD: neural tube defect; Diff: difference between countries for the same period of time; DID: difference-in-difference analysis.

For Chile, all NTD types showed statistically significant pre- vs. post-FAF differences; the greatest post- FAF SR increase was observed for anencephaly, cervico-thoracic spina bifida and cephalocele. Similar results were found for Argentina, with the exception of lumbo-sacral spina bifida which showed no significant SR difference when compared with Venezuela.

**Fig 2:**
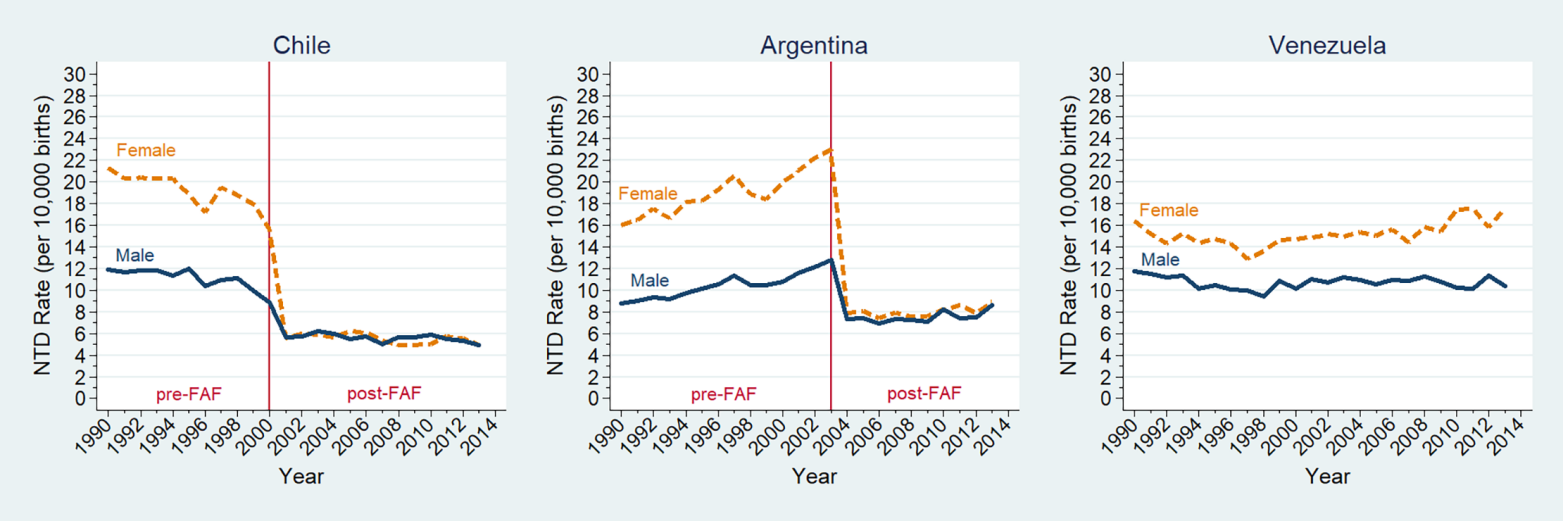
Changes in NTD rates by sex in Chile, Argentina and Venezuela (1990–2013). NTD: neural tube defect; FAF: folic acid fortification; Neural tube defects rates (per 10,000 births) for females (dashed orange line), and males (full blue line). Rates estimated by multivariate regression models adjusted by hospital.

Before FAF in Chile, the NTD prevalence for females was 1.74 times higher than for males. This almost two-fold difference was also observed for anencephaly and spina bifida (total and cervico-thoracic type), while lumbo-sacral spina bifida and cephalocele showed no significant differences. After FAF, the NTD prevalence showed a significant reduction of 49% in males and 70% in females (Table 3); the decline, which was slower in males than in females, could be observed for all NTD types (Fig 2).

**Table 3:** Reduction in NTD rates (per 10,000 births) between pre- and post-FAF periods, by sex and country.

		Pre-FAF				Post-FAF				Reduction (%)	
Country	Defect	Male	95% CI	Female	95% CI	Male	95% CI	Female	95% CI	Male	Female
CHL	NTD total	10.85	9.03–12.93	18.90	16.42–21.67	5.54	4.49–6.76	5.55	4.47–6.81	48.9	70.6
	Anencephaly	4.51	3.37–5.59	8.95	7.26–10.90	2.17	1.53–3.98	2.65	1.93–3.56	51.9	70.4
	Spina bifida	5.03	3.82–6.51	7.95	6.36–9.80	2.45	1.77–3.31	2.29	1.62–3.15	51.3	71.2
	Cervico-thoracic	1.22	0.66–2.04	3.38	2.38–4.65	0.11	0.00–0.41	0.12	0.00–0.44	91.0	96.4
	Lumbo-sacral	3.82	2.78–5.13	4.57	3.39–6.02	2.34	1.68–3.18	2.17	1.52–3.00	38.7	52.5
	Cephalocele	1.30	0.73–2.15	2.01	1.26–3.04	0.92	0.52–1.48	0.60	0.28–1.11	29.2	70.1
ARG	NTD total	10.20	9.32–11.11	18.59	17.37–19.58	7.25	6.09–8.57	7.56	6.34–8.93	28.9	59.3
	Anencephaly	3.82	3.29–4.42	9.50	8.43–10.57	3.04	2.32–3.94	2.81	2.09–3.70	20.4	70.4
	Spina bifida	5.26	4.93–5,95	7.00	6.25–7.81	3.36	2.59–4.33	3.70	2.86–4.69	36.1	47.1
	Cervico-thoracic	1.31	1.01–1.68	2.32	1.91–2.81	0.63	0.32–1.10	0.44	0.19–0.86	51.9	81.0
	Lumbo-sacral	3.95	3.40–4.55	4.67	4.07–5.64	2.73	2.04–3.75	3.25	2.48–4.19	30.9	30.4
	Cephalocele	1.13	0.85–1.47	2.09	1.69–2.55	0.84	0.48–1.37	1.04	0.63–1.63	25.7	50.2
VEN	NTD total	10.81	9.35–12.42	15.14	13.35–17.10	-	-	-	-	-	-
	Anencephaly	3.57	2.75–4.54	5.24	4.21–6.44	-	-	-	-	-	-
	Spina bifida	6.58	5.46–7.87	8.68	7.34–10.18	-	-	-	-	-	-
	Cervico-thoracic	2.41	1.75–3.24	3.20	2.41–4.17	-	-	-	-	-	-
	Lumbo-sacral	4.17	3.28–5.21	5.47	4.42–6.70	-	-	-	-	-	-
	Cephalocele	0.66	0.34–1.15	1.22	0.76–1.87	-	-	-	-	-	-

NTD: neural tube defect; FAF: folic acid fortification.

Similar results were observed in Argentina. Before FAF, the NTD prevalence was 1.82 times higher in females than in males, with the greatest differences for anencephaly and cervico-thoracic spina bifida. After FAF, the NTD prevalence showed significant reductions of 29% in males and 59% in females (Fig 2 and Table 3).

In Venezuela, the prevalence of the whole NTDs and of all its types showed no SR variation, it was always higher in females than in males (Fig 2).

## Discussion

### Sex-specific effects of folic acid on the prevalence of NTDs and changes in their Sex Ratio

We found evidence that in the two countries where a folic acid fortification effect has been demonstrated [1], the NTD prevalence reduction after fortification occurred at a higher rate in females than in males, and that this difference was more evident for anencephaly and spina bifida (total and cervico-thoracic type). Notably, these anomalies are, according to the literature, especially predominant in females.

Our results also showed that the relative increase in the number of males with NTD did not depend on an overall shift of the SR after FAF, as the population SR did not change during the whole pre- and post- fortification periods. Similarly, in a study performed on a Chinese population Zheng et al. [25] showed that the proportion of male births did not rise after periconceptional use of multivitamin supplements containing folic acid.

In 1999, a folic acid campaign for the prevention of NTD started in Nuevo León, Mexico. After two years fortification, a significant rate reduction occurred of overall NTD cases, as well as of the proportion of affected females. Female cases who during 1999 represented 59% of all NTDs, had declined to 37% in 2000, a reduction that was statistically significant for spina bifida and anencephaly, leading to an almost equal distribution by gender in 2001 [19].

Kandasamy et al. [26] reported a decline in overall NTD defects, as well as an increase in the proportion of male cases after preconceptional prescription of folic acid had started. Their sample size was, however, small and included associated anomalies.

Contrarily, in a study on sex ratio and birth defects of 25,952 infants included in the US National Birth Defects Prevention Study, Michalski et al. [3] reported a sex ratio close to one for the whole NTDs, as well as for each of the studied subtypes. However, and as a possible explanation for their results opposing those of others [2, 4–5], most of the affected infants were born after food supplementation with folic acid had started.

Referring to a study carried out in Canada [27], Evans [28] reported additional data on the sex distribution of 2,521 NTD cases before and after folic acid fortification. Before fortification the proportion of female patients with anencephaly and with spina bifida differed significantly from the expected proportion at birth, while after fortification the SR of both defects had "normalized".

### Why does folic acid show a greater protective effect in females than in males?

We believe that the reason most probably relates to the still unanswered question of why are females more often affected by NTDs than males. Many isolated birth defects predominate in one sex over the other, and especially NTDs are among those with the highest SR difference [2]. Although a number of possible causes have been mentioned, such as difference in growth and development rates between male and female embryos, higher prenatal mortality rates in males, epigenetic phenomena and X chromosome inactivation, this issue still remains unsolved.

Furthermore, folic acid is involved in a great number of cell functions [29], hindering the identification of a specific pathway responsible for its differential effect on males and females.

Here we propose some interpretations, based on three theories mentioned as possible causes for the relative female excess in NTDs.

First, in a study on the sex ratio in infants with NTDs, Källén et al. [7] observed a less marked female excess for associated NTDs, and a predominance of males with NTDs lost during early gestation. Both observations would explain the relative predominance of females with isolated NTDs. The greater effect of folic acid on isolated (predominantly female) NTD cases [1] would therefore lead to a relative increase of associated (predominantly male) NTD cases. This theory, however, does not apply to our work which did not include associated NTD cases.

A second hypothesis that could explain the excess of females with NTD refers to the mechanism of X chromosome inactivation [6] through methylation in female embryos, leading to a reduction in the amount of available methyl groups necessary for other functions, including neural tube closure. Since folic acid is involved in the production of the universal methyl donor S-adenosyl-methionine, folate fortification would lead to an increase in the amount of available methyl groups, favouring neural tube closure, preferentially in female embryos.

A similar female excess for NTDs has been observed in mice [6] and here the mentioned mechanism through methylation could also be involved. Okano et al. [30] have shown that the gene Dnmt3b, responsible for de novo methylation of DNA in mouse embryos, is strongly expressed in elevating cranial neural folds, and its null mutation leads to exencephaly. It has already been shown that folic acid is involved in gene transcription. On the one hand by modifying the chromatin structure of certain genes, and on the other it activates folate receptor alpha which, acting as a transcription factor, regulates the expression of a number of genes crucial for embryo development. In mice, disruption of both folate receptor 1 (FOLR1) alleles coding for folate receptor alpha (FRα) resulted in a number of malformations and embryonic death at the time of NT closure [31].

Furthermore, Strandgaard et al. [32] have observed FRα expression in mouse and human ovarian follicles, as well as a high and specific presence of FRα in developing ovaries. They also observed the presence of maternally contributed FRα in the 2-cell stage embryo, and FRα antibodies have been identified in women with recurrent NTD pregnancies [33–35]. The authors concluded that although zygotic FRα is required to foster embryonic development, maternally contributed FRα protein is necessary to sustain it [32].

These findings, indicating a maternal factor in the etiology of NTDs, may explain the higher frequency of female connecting relatives in families with more than one NTD affected individuals [36]. Deak et al. [36] observed a highest proportion of affected among third degree relatives, or mother’s sister’s children than among proband's siblings. They hypothesize about a genetic model including a single dominant gene with reduced penetrance, where the child’s sex, genomic imprinting patterns, methylation status, and folate supplementation could modulate the segregation of NTD in these families [36].

The third theory refers to the multifactorial etiology of NTDs [2, 7, 37] involving both genetic and environmental factors. According to the multiple threshold model [8], NTDs, being more prevalent in females, suggest a double threshold where males, possibly with a greater genetic load than females, are less liable to environmental factors. Evans [28] considered that the proportionately greater reduction of affected females after folic acid fortification could, among other possible explanations, be due to their greater liability to environmental factors.

On the other hand, NTDs are often lumped into and analyzed as one single category, despite their demonstrated etiological heterogeneity [29, 37]. It can be expected that their heterogeneous nature will reflect in a number of characteristics, including different SRs according to the anatomical site of the defect. Data from the literature, as well as our own have not only shown that anencephaly and cervico-thoracic spina bifida have a stronger female predominance than lower defects, but even that the latter could be more frequent in males [38–40]. Nevertheless, a certain homogeneity seems to exist among some subtypes, as suggested by the greater female predominance in anencephaly and cervico-thoracic spina bifida, as well as by their similarly stronger response to folic acid fortification.

To date and to our knowledge this is the first study on the differential effect of folic acid on males and females, using a specifically developed methodology.

We propose that folic acid exerts its protective effect through at least two different mechanisms; because if the mechanism were the same for males and females, the NTD reduction after fortification would probably be similar for both.

One mechanism, acting in females, has a stronger effect, and could perhaps be related to the epigenetic X-inactivation theory. Another or more than one and possibly weaker mechanism may act in both males and females through any of the many other pathways where folic acid is involved. The markedly steeper reduction for females than for males shown in Fig 2 could reflect this differential effect, while the final plateau, at an equal level for both sexes, might represent a remaining group of NTD cases, no longer susceptible to folic acid. It has been estimated that about one third of NTDs may be folic acid resistant [37], which approximately corresponds to the final rate at the plateau level.

### Strengths and weaknesses

This study was developed in the framework of the ECLAMC program, based on a large sample size, standardized forms, and criteria that allowed the comparison between time periods within and between countries. Interviews were conducted by a qualified and experienced team from a large South American hospital-based network. Descriptions of congenital anomalies were reviewed by expert geneticists who established criteria for inclusion and exclusion of cases. Biases in the definition of sex for each case were not expected because of the high observational value of this variable.

Nevertheless, and despite the sample size, we were only able to detect sex ratio changes of at least 25%. Biases in exposure to folic acid were also expected. The information was acquired through an ecological design where ecological fallacies, such as uncertainty regarding the doses used at an individual level, cannot be ruled out. Therefore, we defined two periods, with and without fortification, and incorporated a country without national policies of folic acid fortification as control.

## Conclusions

Up to date and to our knowledge, the sex-specific effect of folic acid on the prevalence of NTDs has not been explored.

Our results indicate that the prevalence of NTDs, mainly anencephaly and cervico-thoracic spina bifida, showed a greater reduction and at a faster rate in females than in males after folic acid fortification, reinforcing the concept of etiological heterogeneity of NTDs.

Furthermore, some of the proposed sex-related mechanisms involved in the prevalence reduction of NTDs might be useful to identify the pathogenesis of these defects as a whole and especially in cases susceptible to the protective effect of folic acid.

## Supporting information

S1 Database Entire database including raw data to replicate all analyses. Number of cases and total births are tabulated by country, year, hospital and sex; and sorted by: "country + year + hospital + sex". (File name: S1_Database.txt; text file delimited by tab; 13 variables; 1,546 registries).
(TXT)

S2 Database Supporting information file. Supporting information file (S2_Database_SuppFile.pdf) with a complete description of database structure and variables.
(PDF)
