# Correction: Neural tube defects: Sex ratio changes after fortification with folic acid

**DOI:** 10.1371/journal.pone.0345341

**Published:** 2026-03-17

**Authors:** Fernando A. Poletta, Monica Rittler, Cesar Saleme, Hebe Campaña, Juan A. Gili, Mariela S. Pawluk, Lucas G. Gimenez, Viviana R. Cosentino, Eduardo E. Castilla, Jorge S. López-Camelo

The first author, Jorge S. López-Camelo, is incorrectly noted as the corresponding author. The correct corresponding author is Fernando A. Fernando A. Poletta’s email address is: fpoletta@cemic.edu.ar.
