# Supplementary material for: Neural tube defects: Sex ratio changes after fortification with folic acid
Source: PLoS One. 2018 Mar 14;13(3):e0193127. doi: 10.1371/journal.pone.0193127 (PMC5851584; doi:10.1371/journal.pone.0193127)
Supplement: S2 Database — Supporting information file (S2_Database_SuppFile.pdf) with a complete description of database structure and variables. (PDF) [file pone.0193127.s002.pdf]

[PONE-D-17-40404]

Supporting Information file

File name: S1_Database.txt

File type: text file delimited by tab.

File structure: Number of variables: 13; Total registries: 1,546. Data (number of cases and total births) are tabulated by country, year, hospital and sex; and sorted by: (country + year + hospital + sex).

Variable name	Type	Description
country	str3	Country: ARG=Argentina; CHL=Chile; VEN=Venezuela
hospital	str5	Hospital IDs: An unique identification alphanumeric code wich is different for each of 95 hospitals from ECLAMC network.
year	str4	Year of birth
sex	byte	Sex: 0=Female; 1=Male
ntd	byte	Ntd: number of cases diagnosed with an isolated neural tube defect.
births	int	Births: Total number of births.
fa_arg	byte	Va_arg: Folic acid fortification period in Argentina: 0=pre-folic acid fortification period (1990-2003); 1=post-folic acid fortification period (2004-2013).
fa_chl	byte	Va_chl: Folic acid fortification period in Chile: 0=pre-folic acid fortification period (1990-2000); 1=post-folic acid fortification period (2001-2013).
anen	byte	Anen: number of cases diagnosed with an isolated anencephaly .
spb	byte	Spb: number of cases diagnosed with an isolated Spina bifida.
cep	byte	Cep: number of cases diagnosed with an isolated Cephalocele.
spb_ct	byte	Spb_ct: number of cases diagnosed with an isolated Cervico-thoracic spina bifida.
spb_ls	byte	Spb_ls: number of cases diagnosed with an isolated Lumbo-sacral spina bifida.
